# A cell wall extract of a *Fusarium incarnatum* strain requires the mitochondrial POLY(A)-SPECIFIC RIBONUCLEASE AtPARN for inducing cytoplasmic calcium elevation in Arabidopsis roots

**DOI:** 10.1007/s12298-025-01600-7

**Published:** 2025-07-02

**Authors:** Y. N. Priya Reddy, Joy Michal Johnson, Ralf Oelmüller

**Affiliations:** 1 Department of Plant Physiology, Matthias Schleiden Institute of Genetics, Bioinformatics and Molecular Botany, Friedrich-Schiller-University, Jena, Germany; 2 Coconut Research Station (Kerala Agricultural University), Balaramapuram, Thiruvananthapuram, Kerala 695501 India; 3 Max Planck Institute for Chemical Ecology, Hans-Knöll-Starße, 07745 Jena, Germany

**Keywords:** *Fusarium*, *Fusarium incarnatum*, Cytoplasmic Ca^2+^ elevation, POLY(A)-SPECIFIC RIBONUCLEASE AtPARN, MALECTIN-DOMAIN CONTAINING CELLOOLIGOMER RECEPTOR KINASE1 (CORK1), *Piriformospora indica*

## Abstract

Cytoplasmic Ca^2+^ ([Ca^2+^]_cyt_) elevation is a rapid response of roots to colonizing beneficial and pathogenic fungi. We have previously demonstrated that the elicitor-active compound cellotriose from a cell wall (CW) extract of the beneficial fungus *Piriformospora indica* requires the MALECTIN-DOMAIN CONTAINING CELLOOLIGOMER RECEPTOR KINASE1 (CORK1) and the mitochondrial POLY(A)-SPECIFIC RIBONUCLASE AtPARN for [Ca^2+^]_cyt_ elevation in Arabidopsis roots. Here, we show that CW extracts from beneficial and pathogenic *Fusarium* strains, in particular *Fusarium incarnatum* strain K23, require AtPARN, but not CORK1 for [Ca^2+^]_cyt_ elevation and the activation of Ca^2+^-dependent downstream responses. [Ca^2+^]_cyt_ elevation by the *F. incarnatum* strain K23 extract does not require the BRASSINOSTEROID INSENSITIVE1-ASSOCIATED RECEPTOR KINASE1 (BAK1) co-receptor or the TWO-PORE Ca^2+^ CHANNEL1 (TPC1) but operates synergistically with the cellotriose- and chitin-induced signaling pathways. We propose a convergence of the signaling pathways induced by the CW extracts from *P. indica* and K23 at AtPARN prior to the increase in [Ca^2+^]_cyt_ ~ 90 s after the stimulus. Furthermore, the elevated [Ca^2+^]_cyt_ levels activate a mild defense response which might be used by the roots to restrict fungal propagation and to balance beneficial and non-beneficial traits in the symbiosis.

**Supplementary Information:**

The online version contains supplementary material available at 10.1007/s12298-025-01600-7.

## Introduction

Cytoplasmic Ca^2+^ ([Ca^2+^]_cyt_) elevation is a rapid response of roots to diffusible signals released by root-colonizing beneficial and pathogenic fungi (Navazio et al. 2007; Johnson et al. 2018; Vadassery and Oelmüller 2009; Tian et al. 2020). The increase in the [Ca^2+^]_cyt_ level is initiated by altered Ca^2+^ channel activity in the root cells. The Ca^2+^ ions are then bound by specific sensor proteins and this process initiates signaling events for the elicitation of specific downstream responses. Root colonizing fungi produce a large variety of elicitor-active compounds, which activate quite different Ca^2+^-dependent responses. Stimuli from pathogenic microbes often activate Ca^2+^ signaling events leading to immune responses (Wang and Luan 2024) while stimuli from beneficial microbes such as arbuscular mycorrhizal fungi induce nuclear Ca^2+^ oscillations in target cells of the host epidermis which lead to the estabishment of a beneficial symbiotic interaction between the two partners (Barker et al. 2017). Therefore, identification of the chemical mediators which are released by the fungi is a prerequisite to elucidate the Ca^2+^-dependent signaling events and downstream responses.

We investigate the symbiotic interaction of the beneficial fungus *Piriformospora indica* (also called *Serendipita indica*) with Arabidopsis roots. The well investigated endophytic fungus interacts with the roots of almost all tested plant species and thus became a model system for beneficial symbiotic interactions (Perez-Alonso et al. 2020; Khalid et al. 2019; Xu et al. 2018; Boorboori and Zhang 2022; Saleem et al. 2022). The fungus releases the elicitor-active compound cellotriose which induces [Ca^2+^]_cyt_ elevation in Arabidopsis root cells (Johnson et al. 2018; Oelmüller 2018). Since the role of cellotriose for the symbiotic interaction is not clear, we investigated the Ca^2+^-induced signaling events in the host. Cellotriose is perceived by the MALECTIN-DOMAIN CONTAINING CELLOOLIGOMER RECEPTOR KINASE1 (CORK1, also called IGP1, IMPAIRED IN GLYCAN PERCEPTION 1) at the plasma membrane of Arabidopsis root cells (Tseng et al. 2022a; Martin-Dacal et al. 2023; Oelmüller et al. 2023). Isothermal titration calorimetry assays showed that the purified ectodomain of CORK1/IGP1 binds cellotriose with high affinity (Martin-Dacal et al. 2023). CORK1 is mainly expressed in the vascular tissue of the upper, fully developed part of the roots which is consistent with the specific interaction of *P. indica* with roots (Gandhi et al. 2024). CORK1-induced responses interfere with chitin-triggered immune responses and are influenced by BRASSINOSTEROID INSENSITIVE 1-ASSOCIATED RECEPTOR KINASE1 (BAK1) and the receptor kinase FERONIA (Gandhi et al. 2024). Both kinases are rapidly phosphorylated by cellotriose in Arabidopsis roots in a CORK1-dependent manner (Tseng et al. 2022a). Cellotriose and other cellooligomers are also generated during the breakdown of cellulose of the plant cell wall (CW). Since CW integrity maintenance is central for plant cells, accumulation of cellotriose might activate an alarm system to induce appropriate downstream immune responses (Tseng et al. 2022a). Microbial interactions can alter or destroy the structure of the plant CW, connecting CW integrity maintenance to immune responses (Tseng et al. 2022a). These observations suggest that cellotriose activate immune responses in root cells. Downstream of CORK1, the POLY(A)-SPECIFIC RIBONUCLEASE AtPARN is required for the [Ca^2+^]_cyt_ response (Johnson et al. 2018). Poly(A)-specific ribonucleases influence the poly(A) status of cytoplasmic mRNA in many eukaryotes, and AtPARN has been proposed to participate in mRNA degradation, presumably by shortening the poly(A)-tail of specific mRNA species (Chiba et al. 2004; Nishimura et al. 2005, 2009; Johnson et al. 2018). AtPARN has been linked to phytohormone functions and partial loss of PARN activity affects abscisic acid and salicylic acid metabolism (Nishimura et al. 2009). Furthermore, AtPARN directly regulates the poly(A) tract of mitochondrial mRNA in conjunction with a bacterial-type poly(A) polymerase in Arabidopsis (Hirayama et al. 2013). The role of AtPARN in [Ca^2+^]_cyt_ elevation in response to fungal elicitor-active compounds is enigmatic. Otsuka et al. (2021) demonstrated that mitochondrial RNA processing is linked to lateral root morphogenesis. Analysis of temperature-dependent fasciation mutants of Arabidopsis demonstrated that mitochondrial RNA processing is required for limiting cell division during early lateral root organogenesis. Whether this control mechanism involves Ca^2+^ signaling, has not yet been investigated (cf. Discussion). Here, we demonstrate that besides *P. indica* also CW preparations from various pathogenic and beneficial *Fusarium* strains require PARN, but not CORK1, for [Ca^2+^]_cyt_ elevation in Arabidopsis roots. Although there are remarkable differences among the tested *Fusarium* strains, the results support the important role of PARN for [Ca^2+^]_cyt_ elevation induced by CW-derived fungal elicitors. In particular, [Ca^2+^]_cyt_ elevation by a recently characterized *Fusarium* strain K23 is completely dependent on PARN. Recently, it has been shown that the fungus plays an important role in salt stress tolerance. K23 alleviates salt stress in *A. thaliana* through its root hair growth promoting effect (Onejeme et al. 2024), and combined -omics approaches revealed that induction of root hair growth by K23 is a prerequisite for the enhanced salt stress tolerance. The authors identified the NAC transcription factor JUNGBRUNNEN1 (JUB1) as a target of K23 colonisation. Stimulation of *JUB1* expression by the fungus leads to the repression of gibberellin biosynthesis which in turn contributes to sustained root hair growth. Furthermore, Pallavi et al. (2024) investigated the role of K23 for salt stress tolerance in tomato. A combined transcriptomics and proteomics approach demonstrated that colonization of tomato by K23 results in an extreme reprogramming of the expression and proteome profiles of tomato seedlings under salt stress. How early signaling events such as [Ca^2+^]_cyt_ elevation in response to fungal elicitors are related to the better performance of the hosts under abiotic stress is not known (cf. Discussion).

## Methods and materials

### Growth of arabidopsis and fungi, co-cultivation

*A. thaliana* seeds were surface-sterilized for 8 min in sterilization solution containing lauryl sarcosine (1%) and Clorix cleaner (23%). Surface-sterilized seeds were washed with sterilized water 8 times and placed on Petri dishes with MS medium supplemented with 0.3% gelrite (Murashige and Skoog 1962). After cold treatment at 4 °C for 48 h, plates were incubated at 22 °C under long day conditions (16 h light/8 h dark; 80 μmol m^−2^ s^−1^).

The aequorin-containing wild-type (WT) [pMAQ2] line (AeqWT; Knight et al. 1991), an ethyl methane sulfonate-induced mutant lines for CORK1 (At1g56145) and the POLY(A)-SPECIFIC RIBONUCLEASE AtPARN (At1g55870), the *bak1* and *two-pore Ca*^*2*+^ *channel1* (*tpc1*) mutants in AeqWT background (Tseng et al. 2022a; Johnson et al. 2018) were used in this study.

The *Alternaria brassicae* (FSU-3951), *Mortierella hyalina* (FSU-509) and various *Fusarium* strains (cf. Table 1) were obtained from Jena Microbial Resource Center (Jena, Germany). The *F. incarnatum* strain K23 was obtained from Dr. Nataraja (University of Agricultural Sciences, Bengaluru, India) and described in Pallavi and Nataraja (2022) and Pallavi et al. (2024). The *Trichoderma confertum*-related strain, as well as the growth conditions for all strains were described in Tseng et al. (2020, 2022a). For physiological experiments, they were cultured and maintained on Kaefer medium (KM), pH 6.5, as described by Johnson et al. (2011b). *P. indica* (Johnson et al. 2018) was inoculated to *Arabidopsis* seedlings and re-isolated from the infected tissues every 6 months (Johnson et al. 2013). Co-cultivation occurs with a fungal plaque which was positioned approximately 1 cm away from the roots on KM medium in Petri dishes. Co-cultivation was performed for 7 days until harvest on day 12. The seedlings for the root colonization assays (Table 2) were grown in 20-cm high jars on solid KM, pH 6.5, under the same condition as the seedling grown in Petri dishes, and the time points of the harvest are indicated in the figures.

**Table 1:** PARN is required for [Ca^2+^]_cyt_ elevation induced by CW extracts from *Fusarium* strains in Arabidopsis roots

*Fusarium spp*	Strain collection no	Reduction (%)
*F. acuminatum*	SF004462	45.9
*F. sporotrichioides*	SF005203	48.8
	SF005204	53.5
*F. langsethiae*	SF005397	47.6
	SF005462	38.9
*F. poae*	SF005063	60.8
	SF005395	48.9
	SF005396	37.5
	SF005466	60.8
	SF005469	-3.8
*F. graminearum*	SF005195	44.4
	SF005206	40.0
	SF005207	25.0
	SF005402	27.0
	SF005465	13.5
*F. culmorum*	SF005058	37.5
	SF005400	46.8
	SF005522	48.6
*F. incarnatum*	SF010308	41.0
	K23	100.0
*F. equiseti*	SF005061	42.9
	SF005430	42.5
	SF005459	20.6
	SF012324	41.2
*F. avenaceum*	SF005057	37.5
	SF005251	28.6
*F. lateritium*	SF001374	47.5
	SF005242	57.1
*F. verticillioides*	SF002279	15.0
	SF006225	12.5
	SF006226	31.7
*F. solani*	SF001283	37.5
	SF011443	16.7
*F. proliferatum*	SF005069	28.6
*F. lycopersici*	SF003853	41.5
*F. oxysporum*	SF003753	18.2
	SF004818	5.7
	SF005060	40.0
	SF013969	32.5

**Table 2:** Root colonization of WT, *cork1* and *parn* plants between 5 and 25 days after infection

Days after infection	WT	*cork1*	*parn*
5	1.0 ± 0.3	1.2 ± 0.4	0.9 ± 0.3
10	1.9 ± 0.5	2.6 ± 0.7	3.3 ± 0.5*
15	2.5 ± 0.4	3.9 ± 1.3*	4.1 ± 0.9
25	2.7 ± 0.7	4.3 ± 1.5	5.6 ± 1.4*

### CW preparations

The CW extracts were prepared using the protocol of Anderson-Prouty and Albersheim (1975) with modifications. Mycelia from 14-day-old liquid cultures were homogenized using mortar and pestle in 5 mL water g^−1^ mycelium. The homogenate was filtered using a coarse sintered glass funnel. The residue was washed three times with water, once with chloroform/methanol (1:1) and finally in acetone. This preparation was air dried for 2 h and the mycelial CW material was recovered. Elicitor fractions were prepared from mycelial CWs by suspending 1 g of CW in 100 mL water and autoclaving for 20 min at 121 °C. The suspension was centrifuged at 28,000 g for 10 min, filter-sterilized using a 0.22 μm filter and concentrated to half before further assay.

### Reactive oxygen species (ROS) and [Ca^2+^]_cyt_ measurements

Seedlings were grown on Hoagland agar medium (Hoagland’s No. 2 Basal Salt Mixture; Sigma-Aldrich) for 12 days before harvesting approximately 70% of the roots for ROS and [Ca^2+^]_cyt_ measurements (Vadassery et al. 2009; Vadassery and Oelmüller 2009; Johnson et al. 2011a, b).

For ROS measurement, root tissue was incubated in sterile water in a 96-well plate in the dark at room temperature for 1 h. Prior to the elicitor treatment, water was replaced by 150 μL of assay solution containing 2 μg/mL horse radish peroxidase (Sigma-Aldrich) and 100 μM luminol (FUJIFILM Wako Chemicals Europe GmbH, Neuss, Germany).

The [Ca^2+^]_cyt_ concentration was inferred from aequorin-based luminescence (Knight et al. 1991). Root tissues were incubated overnight in 150 μL of 7.5 μM coelenterazine solution (P.J.K. GmbH, Kleinblittersdorf, Germany) in a 96-well plate in the dark at room temperature. For the comparative analyses of CW extracts from different fungal species (cf. Table 1), the amounts of the CW extracts, which give the same [Ca^2+^]_cyt_ elevation response in AeqWT roots were first determined. This amount was then used to test the [Ca^2+^]_cyt_ response in the AeqWT mutant in the *parn* background. It is worth mentioning that neither the amounts of the CW extracts from the different fungal species nor the shape of the [Ca^2+^]_cyt_ response curves (maximal [Ca^2+^]_cyt_ response 90 s after application of the extracts to the roots) differed significantly among the different fungal extracts. This is likely due to the highly optimized and standardized CW extraction procedure applied to the different fungal species.

Bioluminescence counts from elicitor application were recorded as relative light units (RLU) with microplate luminometer (Luminoskan Ascent version 2.4, Thermo Fisher Scientific or Mithras LB940, Berthold Technologies, Bad Wildbad, Germany).

Cellotriose (C1167, Sigma-Aldrich, or 0-CTR-50MG, Megazyme, Wicklow, Ireland), flagellin22 (flg22) (QRLSTGSRINSAKDDAAGLQIA, Biosynth Laboratories, Billingham, Cleveland, USA) and chitin (OH07433, Carbosynth, Berkshire, United Kingdom) were used as elicitors (10 μM), as described previously (Johnson et al. 2018).

### RNA extraction and qPCR

Root tissue was homogenized in liquid nitrogen. RNA extraction was performed with Trizol™ reagent (Thermo-Fisher Scientific), treated with Turbo DNA-free™ Kit (Thermo-Fisher Scientific), and reverse transcribed with Revert Aid Reverse Transcriptase (Thermo-Fisher Scientific) according to the manufacturer's instructions.

Quantitative reverse transcription PCRs (RT-qPCRs) were performed with Dream Taq DNA Polymerase (Thermo-Fisher Scientific, Germany) with the addition of Evagreen^®^ (Biotium, Fremont, California, USA). CFX Connect™ Real-Time PCR Detection System (Bio-Rad, Feldkirchen, Germany) was used for running and analyzing qPCRs. The expression of genes was normalized to the housekeeping gene encoding a ribosomal protein (RPS; At1g34030). The resulting ΔCq values were used for statistical analysis. All primers used are listed in Supplementary Table S1. For the quantification of root colonization, the *P. indica PiTEF1* transcript level was determined relative to the Arabidopsis *AtRPS* transcript level in roots.

## Results

### CW preparations from ***Fusarium incarnatum*** strain K23 require AtPARN, but not CORK1 for [Ca^2+^]_cyt_ elevation

**Fig. 1:**
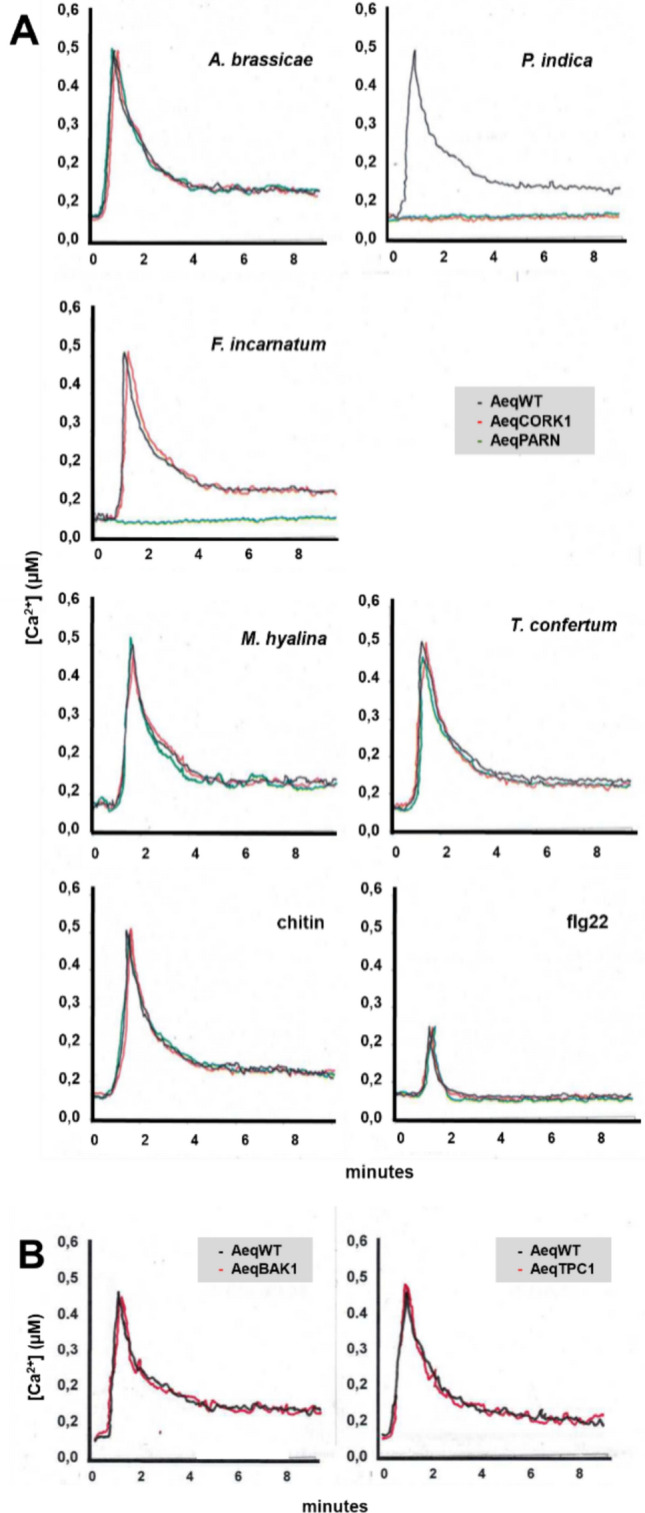
**A** [Ca^2+^]_cyt_ elevation in Arabidopsis roots induced by CW extracts from pathogenic and beneficial fungi, as well as chitin and flg22. The panels show the responses in WT, *cork1* and *parn* roots. Each line is based on more than 10 measurements. **B** *F. incarnatum* strain K23-induced [Ca^2+^]_cyt_ elevation does neither require BAK1 nor TPC1

A CW preparation of the *F. incarnatum* strain K23 induced rapid [Ca^2+^]_cyt_ elevation, comparable to the CW preparations from *P. indica* and *Alternaria brassicae*. The [Ca^2+^]_cyt_ response induced by a CW preparation from the pathogen *A. brassicae* is comparable in WT, *cork1* and *parn* roots (Fig. 1A). In contrast, the *P. indica*-induced response required CORK1 and PARN. The CW preparation from *F. incarnatum* strain K23 required PARN, but not CORK1 (Fig. 1A). This indicates that the elicitor-active compound(s) from *F. incarnatum* strain K23 is probably not a cellooligomer, and the signal transduction pathways induced by the CW extracts from the two beneficial fungi *F. incarnatum* strain K23 and *P. india* leading to [Ca^2+^]_cyt_ elevation converge at AtPARN. Chitin and the CW extracts from two other beneficial fungi, *Mortierella hyalina* (Johnson et al. 2019) and a *Trichoderma confertum*-related strain (Tseng et al. 2020, 2022b) induced comparable [Ca^2+^]_cyt_ elevations in the WT, *cork1* and *parn* mutants. The same was observed for flg22, although the response in the roots is quite low (Fig. 1A).

### *F. incarnatum* strain K23-mediated [Ca^2+^]_cyt_ elevation in Arabidopsis roots does not require the BAK1 co-receptor or the Ca^2+^channel TPC1

BAK1 is a co-receptor for FLAGELLIN SENSING2 (FLS2) and ETHYLENE RESPONSE FACTOR (ERF) (Chinchilla et al. 2007), and it is believed that CHITIN ELICITOR RECEPTOR KINASE1 (CERK1) signaling does not require BAK1, although there might be cross-talks between the pathways (Gong et al. 2019). Activation of the receptor kinases FLS2, ERF and CERK1 results in rapid [Ca^2+^]_cyt_ elevation (Jeworutzki et al. 2010, Johnson et al. 2018). Since the active compound in the *F. incarnatum* strain K23 CW extract is likely a oligo- or polysaccharide (cf. Methods and Materials), we tested whether [Ca^2+^]_cyt_ elevation induced by the K23 CW extract requires BAK1. Figure 1B shows that the [Ca^2+^]_cyt_ response in WT and the *bak1* mutant is comparable. Furthermore, TPC1 has been demonstrated to be involved in establishing [Ca^2+^]_cyt_ homeostasis (Schonknecht 2013), but the *tpc1* mutant also showed a wild-type response. We conclude that both proteins are not involved in *F. incarnatum* strain K23-induced [Ca^2+^]_cyt_ elevation.

### The CW extract from *F. incarnatum* strain K23 operates synergistically with cellotriose and chitin

**Fig. 2:**
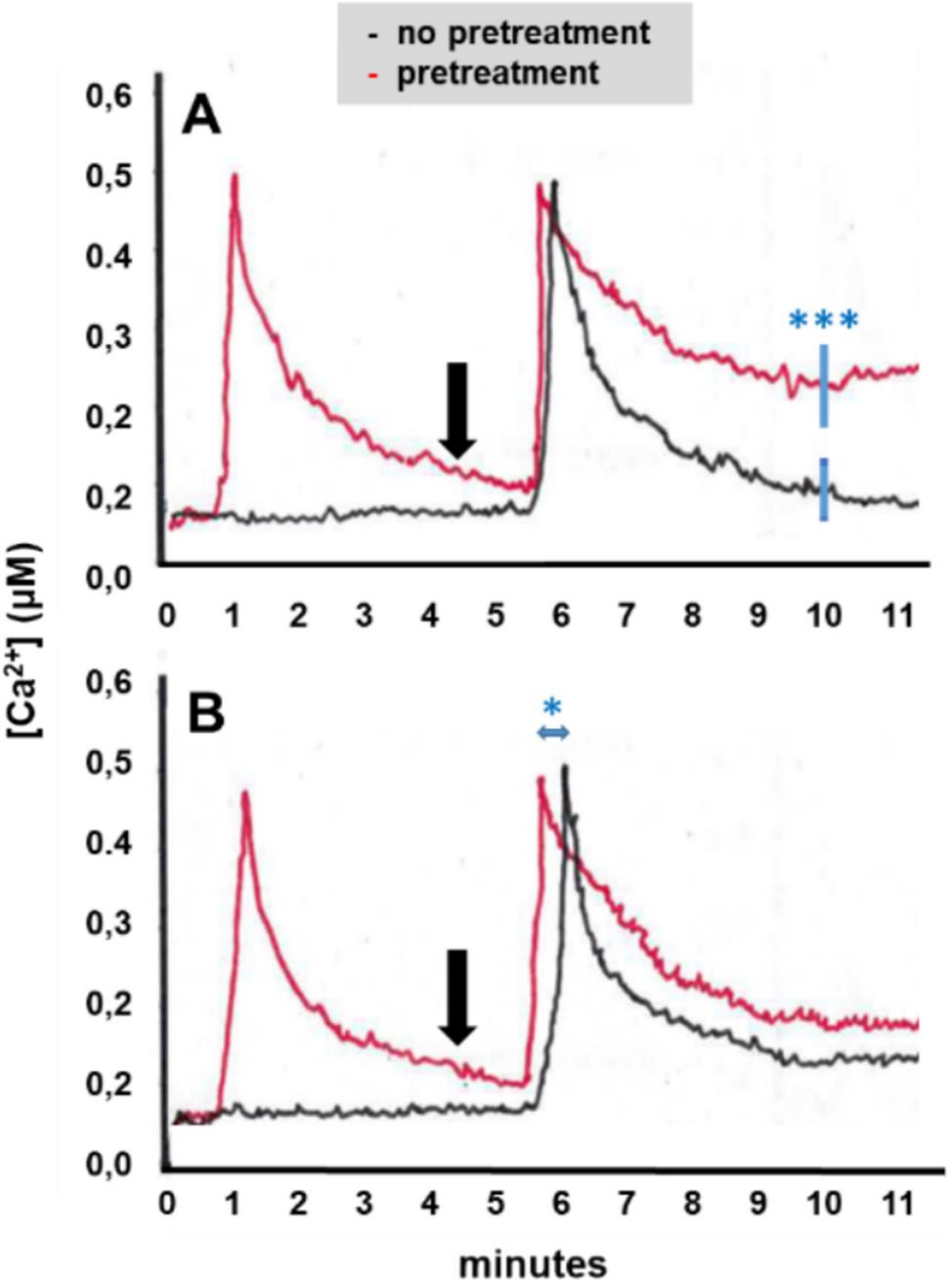
Arabidopsis roots were first exposed to cellotriose **A** or chitin **B** and 4.5 min later (indicated by black arrow) to the CW extract of *F. incarnatum* strain K23 (red). Control roots did not receive the cellotriose or chitin pretreatment (black). Based on ten independent experiments, *** in **A** indicates significant difference at the 10 min timepoint, * in **B** between appearance of peak maxima for pretreated and non-pretreated roots

Next, we tested whether signaling induced by cellotriose from *P. indica* cross-talks to signaling induced by the CW extract from *F. incarnatum* strain K23*.* Aequorin WT seedlings were first treated with cellotriose and 4.5 min later with the CW extract from K23. In ten independent experiments, we observed that the [Ca^2+^]_cyt_ response to the K23 stimulus lasted longer when a cellotriose stimulus was given before the K23 stimulus (Fig. 2A). A possible explanation could be that signaling around or downstream of PARN becomes activated by cellotriose and that the active state allows a longer lasting increase of Ca^2+^ into the cell, once a second stimulus with the CW extract from K23 is given (Fig. 2A). In the reverse experiment (first application of the CW extract of K23 followed by cellotriose application), we did not observe significant differences in the cellotriose-induced [Ca^2+^]_cyt_ elevation with or without the pre-treatment (data not shown).

Furthermore, since CERK1 responds to treatments with the oligosaccharide chitin, we tested whether there is a synergistic effect between chitin signaling and signaling induced by the CW extract of *F. incarnatum* strain K23. Aequorin WT seedlings were first treated with chitin and 4.5 min later with the CW extract from K23. In ten independent experiments, we observed that the second stimulus induced faster [Ca^2+^]_cyt_ elevation (Fig. 2B). The difference between the two [Ca^2+^]_cyt_ peaks was 15.6 ± 5.1 s (n = 10). Similar to the results with the cellotriose pretreatment, the decline in the [Ca^2+^]_cyt_ level was slower compared to the decline in seedlings without chitin pretreatment, however the differences were not significant. Taken together the results suggest that early signaling events induced the *F. incarnatum* strain K23 CW extract crosstalk with those induced by cellotriose and chitin.

### [Ca^2+^]_cyt_ elevation is required for stimulation of ROS production and *WRKY30* expression

**Fig. 3:**
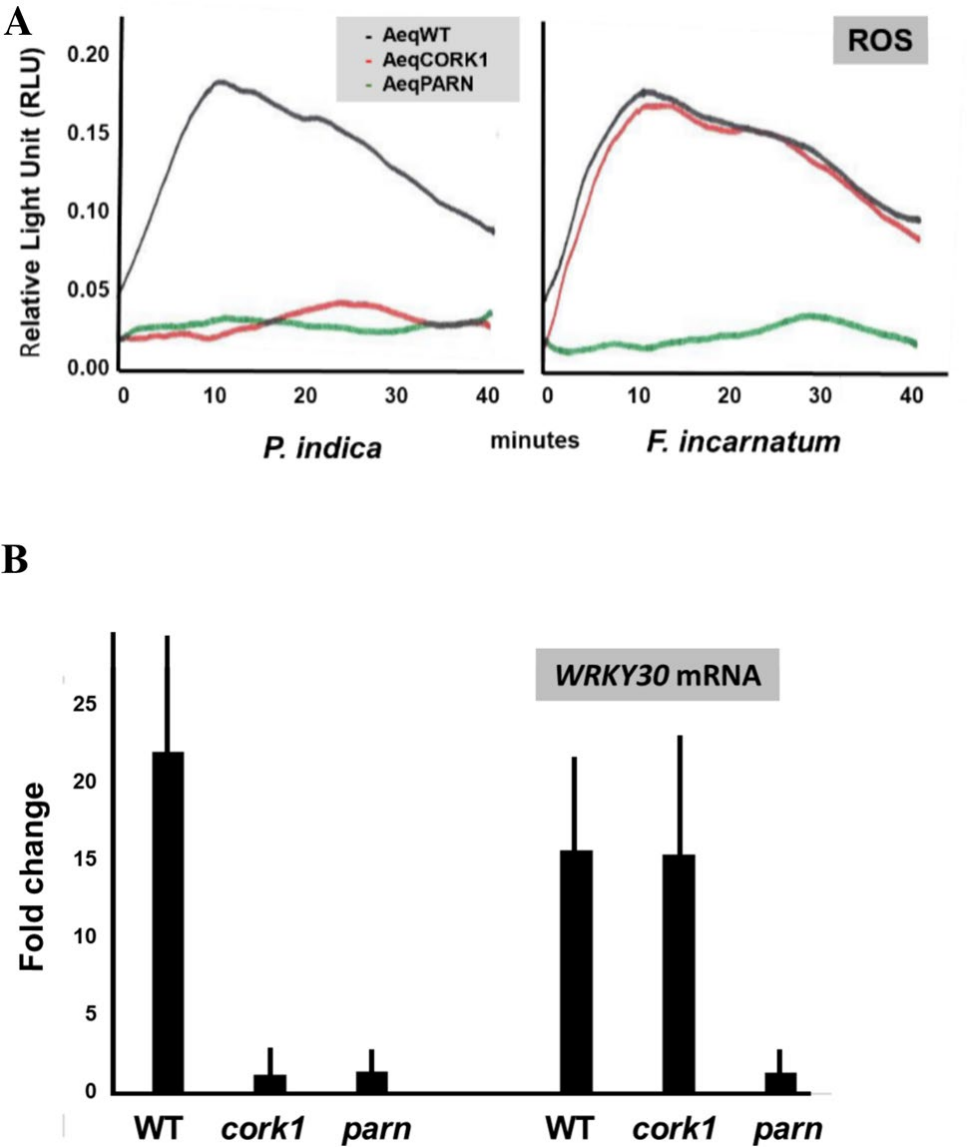
Stimulation of ROS production (**A**) and *WRKY30* expression (**B**) in response to the *P. indica* and *F. incarantum* strain K23 CW extracts in Arabidopsis roots. In **A** the aequorin lines used to measure ROS production in response to CW extracts from *P. indica* and the *F. incarantum* strain K23 are indicated. For normalization of the mRNA data in (**B**), cf. Methods and Materials. Based on 3 independent experiments, error bars in (**B**) represent SEs

We have previously shown that [Ca^2+^]_cyt_ elevation in response to cellotriose is required for the stimulation of ROS production and *WRKY30* expression (Johnson et al. 2018; Tseng et al. 2022a). The same holds true for the *F. incaratum* CW extract. When applied to WT and *cork1* roots, a massive stimulation of ROS production and a > tenfold increase in the *WRKY30* mRNA level was detected. These responses could not be detected in the *parn* mutant (Fig. 3). These results suggest that stimulation of ROS production and induction of *WRKY30* expression by the *F. incarnatum* strain K23 CW extract depend on PARN. The CW extract from *P. indica* was used as control. As shown previously (Tseng et al. 2022a; Johnson et al. 2018), stimulation of both responses by cellotriose requires CORK1 and PARN (Fig. 3).

### PARN requirement for [Ca^2+^]_cyt_ elevation differs in different ***Fusarium*** strains

To test whether the PARN required for [Ca^2+^]_cyt_ elevation induced by the *F. incarnatum* strain K23 is strain-specific or a general feature of *Fusarium* fungi, we tested different pathogenic, beneficial and mutualistic *Fusarium* strains. Table 1 shows that almost all strains require PARN for [Ca^2+^]_cyt_ elevation, although the requirements were quite different for the different strains. We did not observe an obvious relationship between the PARN requirement for [Ca^2+^]_cyt_ elevation and the phylogenetic relations of the strains. For instance, [Ca^2+^]_cyt_ elevation induced by the CW extracts from strain SF005469 of *F. poae* does not require PARN, while [Ca^2+^]_cyt_ elevation induced by CW extracts from the two *F. poae* strains SF005063 and SF005466 was more than 60% reduced in the *parn* mutant. The elicitor-active compound in the CW preparations might be present in different concentrations in the investigated *Fusarium* strains, or are structurally different. It is also likely that the CW preparations contain more than one elicitor-active chemical compound. This might explain the differences for the PARN requirement for the induction of [Ca^2+^]_cyt_ elevation.

### Role of Ca^2+^ signaling in the interaction between Arabidopsis and ***P. indica***

The CW extracts which induce [Ca^2+^]_cyt_ elevation derive from beneficial fungi, which raises the question of the function of these elicitor-active compounds and the induced signaling pathways in the symbiotic interaction. Furthermore, which genes are induced in response to [Ca^2+^]_cyt_ elevation in the host? Since CORK1 and PARN are preferentially expressed in roots (Johnson et al. 2018; Gandhi et al. 2024), it is likely that the function associates with the colonization of this organ.

**Fig. 4:**
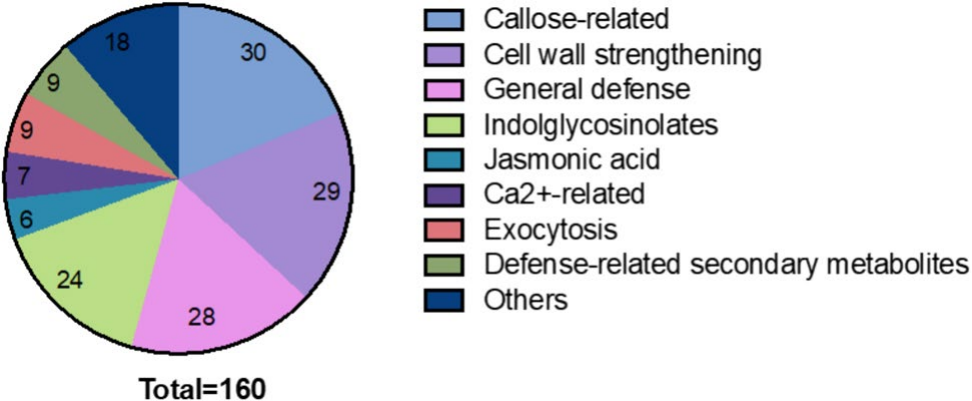
Pie diagram for the 36% defense-related genes among all common genes which are induced more than fourfold by cellotriose in WT roots, but not in the roots of the *cork1* and *parn* mutants

We took advantage of available expression profile data for *P. indica* (Johnson et al. 2018 for the *parn* mutant and Tseng et al. 2022a for the *cork1* mutant), and identified common genes which are induced more than fourfold by cellotriose from *P. indica* in WT roots, but not in the roots of the *cork1* and *parn* mutants. Annotation of the gene products uncovered that 36% of the common genes code for proteins involved in plant defense. Closer inspection of these defense-related genes demonstrated that they are involved in callose deposition and CW thickening, followed by secondary metabolite including indole glucosinolate biosyntheses, Ca^2+^ signaling, exocytosis and jasmonate signaling (Fig. 4). This suggests that the roots perceive the chemical compounds from the colonizing fungi to restrict root colonization and entry of the microbes into the root cells.

To test this idea, we performed colonization studies with *P. indica* in the roots of WT, *cork1* and *parn* mutants. Although not always significant, Table 2 shows that the roots of the *cork1 and parn* plants become more colonized than those of the WT, and this becomes more obvious after longer cocultivation periods.

## Discussion

Our data demonstrate that PARN is not only required for [Ca^2+^]_cyt_ elevation induced by the CW extract from *P. indica*, but also from almost all tested *Fusarium* strains (Fig. 1, Table 1). The requirement of PARN for [Ca^2+^]_cyt_ elevation in response to external stimuli is still enigmatic, however, this study demonstrates that the ribonuclease might be an important signaling molecule in immune responses in roots (cf. below). PARN belongs to the DEED superfamily of deadenylases (Pavlopoulou et al. 2013) and participates in the maturation of RNAs in human and animals (Lee et al. 2019). In animals, yeast and insects, PARN mediates trimming of the poly(A) tails of about 2% of all mRNAs. This affects the stability of these mRNAs and PARN controls the quality of gene expression posttranscriptionally in the cytoplasm (Godwin et al. 2013). In addition, PARN controls the maturation of a class of small RNAs in the cytoplasm of mammal cells (summarized in Godwin et al. 2013). Since the plant PARNs lacks two important domains (R3H and RRM) which are highly conserved in non-plant PARNs and which are important for their function, it is likely that the plant enzyme differs from those in other kingdoms (cf. Godwin et al. 2013). In plants, PARN removes the poly(A) tails of mitochondrial mRNAs (Hirayama et al. 2013; Hirayama 2014, 2021; Kanazawa et al. 2020), and in contrast to human and animal PARNs, the Arabidopsis PARN is located in mitochondria (Hirayama et al. 2013, Waltz et al. 2019). Polyadenylation of the 3´ends of plant mitochondrial mRNAs functions as a degradation mark as part of an mRNA quality and/or quantity control mechanism and removal of the poly(A) tail from mitochondrial mRNAs by PARN is essential for plant mitochondrial function (Hirayama 2021). Since complete knockout lines of the single copy gene *PARN* in Arabidopsis are lethal (Chiba et al. 2004; Reverdatto et al. 2004), functional analyses are often performed with plants with weak mutational allels which show milder but often pleiotrophic effects. For the *parn* mutant with a point mutation used in this study, growth inhibition and pleiotrophic effects are mainly visible for adult plants (Johnson et al. 2018), therefore this line might be a good candidate for further studies.

Impairments on plant mitochondria have severe consequences for cytoplasmic functions and root development. Mitochondria are not only involved in energy and metabolite production but also in cellular processes such as apoptosis, hormone functions and responses to stress (Galluzzi et al. 2012; Liberatore et al. 2016; Hirayama 2021). For instance, partial loss of PARN activity affects abscisic acid and salicylic acid metabolism and signaling which might be a downstream effect of impaired mitochondrial activities. Genetic analyses showed that the hormone effects are independent results in weak *parn* mutants (Nishimura et al. 2009; Hirayama et al. 2013). Libertore et al. (2016) proposed that mitochondria regulate key cytoplasmic players that integrate signals for plant growth and stress responses via early signaling and modulating. SnRK1 (SUCROSE-NON-FERMENTING-1-RELATED PROTEIN KINASE1) in plants and TOR (TARGET OF RAPAMYCIN) in animals are two main players acting towards energy conversion by controlling growth and metabolic reprogramming in response to stress (Lastdrager et al. 2014; Tomé et al. 2014). Key players that are controlled by mitochondria are Ca^2+^, plant hormones, the carbon and metabolic status and ROS (Libertore et al. 2016). Also, Otsuka et al. (2021) suggested that ROS produced by deteriorated mitochondria could be a major factor leading to abnormal root morphogenesis. This supports the idea that major cytoplasmic functions and root developmental programs are impaired in mutants with impaired poly(A) trimming in mitochondria.

Otsuka et al. (2021) showed that temperature-dependent fasciation mutants of Arabidopsis with lesions in mitochondrial RNA processing are impaired in cell division during early lateral root organogenesis. One of the identified mutated proteins, the mitochondrial localized ROOT REDIFFERENTIATION DEFECTIVE1 (RRD1) shows high similarities to PARN. Therefore, it is conceivable that impairments in mitochondrial RNA processing might also control [Ca^2+^]_cyt_ elevation in response to fungal signals. This could occur through a direct signaling pathway (e.g. from the mitochondria to the regulated Ca^2+^ channels) or be the result of energetic/metabolomic changes due to impaired mitochondrial functions. An overlap of signaling components activated by these mutants and root-interacting beneficial fungi is not surprising since both integrate signals for growth and defense (cf. below).

**Fig. 5:**
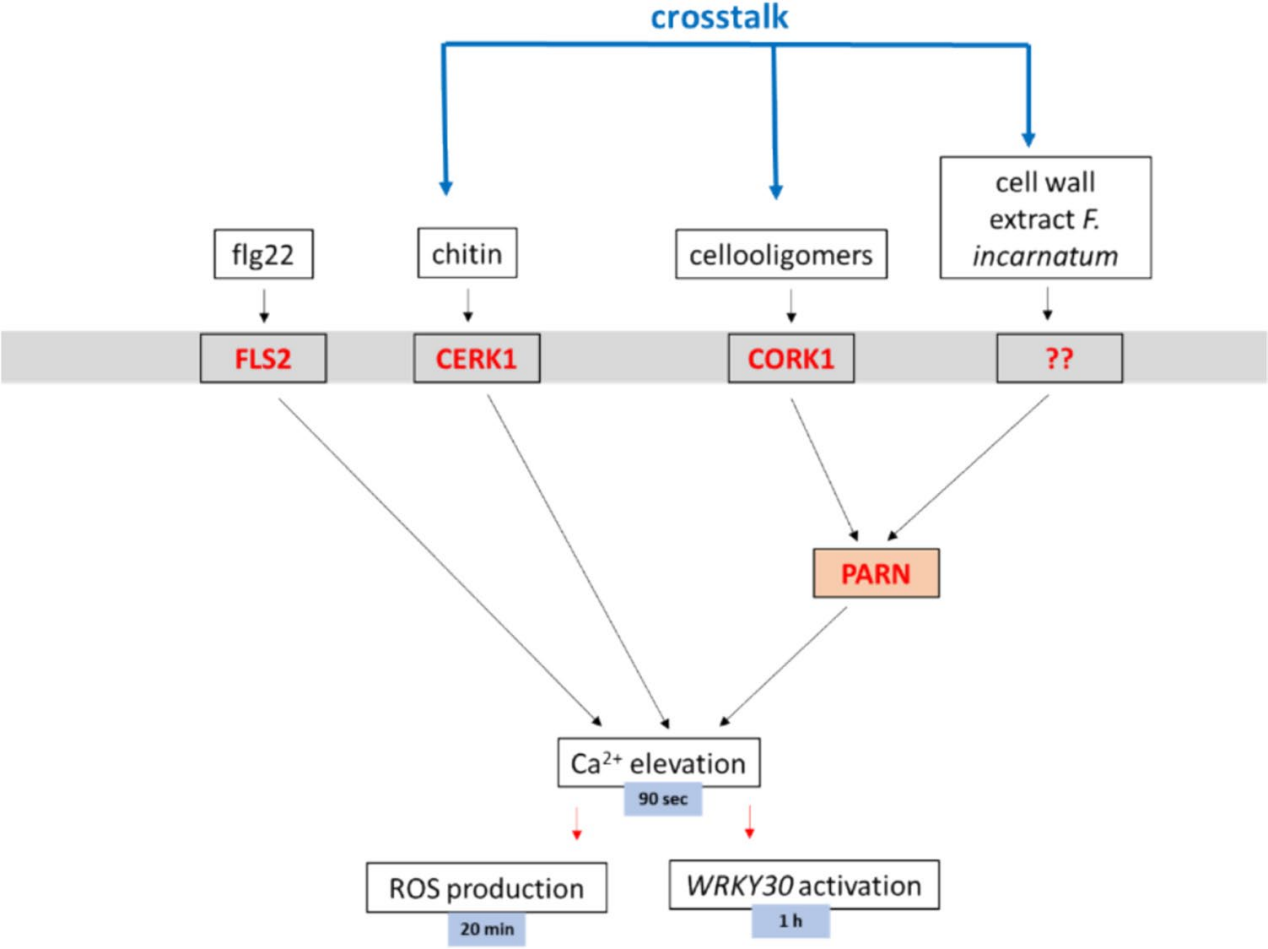
A model describing the signaling events induced by flg22, chitin, cellooligomers and the CW extract from *F. incarnatum* strain K23 in Arabidopsis roots. The latter two stimuli require PARN for the three measured downstream responses, cellooligomers require CORK1 and PARN and flg22 and chitin operate independently. The proposed crosstalk between the activated signal pathways is indicated

We showed that cellotriose from the CW extract of *P. indica* and CW extracts from quite diverse beneficial and pathogenic *Fusarium* strains requires PARN for the induction of [Ca^2+^]_cyt_ elevation. Several other beneficial or pathogenic fungal species which have been tested, do not require PARN for [Ca^2+^]_cyt_ elevation (Fig. 1; Johnson et al. 2019) indicating some specificity. Moreover, the PARN requirement differs substantially among the different *Fusarium* strains. For instance, the CW preparation from the strain SF005469 does not require PARN for [Ca^2+^]_cyt_ elevation, whereas the [Ca^2+^]_cyt_ elevation response induced by the CW preparation from the strain K23 depends completely on PARN. Since the elicitor-active compound(s) are not known at present, this might have different reasons. The different strains might contain different amounts of the same active compound in their CW preparations, different strain might contain different active compounds, or more than one compound is involved in [Ca^2+^]_cyt_ elevation. A major task for the future will be the identification of the elicitor-active compound(s) in the different *Fusarium* strains. Genetic evidence demonstrates that the elicitor-active compound(s) in the *Fusarium* strains is different from cellotriose from *P. indica* (Johnson et al. 2018) since it does not require CORK1 for function. Since CORK1 binds cellooligomers, it is unlikely that the chemical mediator(s) in the *Fusarium* strains is a cellooligomers. Furthermore, [Ca^2+^]_cyt_ elevation is also induced by the pathogen-associated molecular patterns chitin and flg22, but they do not require CORK1 or PARN (Fig. 3). Based on these observations, a model is presented which describes the signaling events induced by cellotriose, the CW extracts from the tested *Fusarium* strains, as well as the two pathogen-associated patterns (Fig. 5). The cellotriose-induced pathway and the pathway induced by the CW extracts from *F. incarnatum* strain K23 converge at PARN, while those of the two pathogen-associated molecular patterns operate independently.

Our data suggests a cross-talk between the signaling events induced by the elicitor-active compound(s) from *F. incarnatum* strain K23, cellotriose and chitin. However, this conclusion might be over-interpreted, since it is based on results obtained with two chemicals (cellotriose and chitin) and a crude CW preparation, from which the ingredients are not known. Cross-talks between signaling pathways occur regularly in all organisms and Gandhi et al. (2023) showed that CORK1 activation by cellotriose altered the phosphorylation pattern of several signaling proteins of other pathways including CERK1 (Gandhi et al. 2023) which results in an alteration of its physiological behavior (Gandhi et al. 2024). Therefore, it is likely, that also the *F. incarnatum* strain K23-induced [Ca^2+^]_cyt_ elevation in Arabidopsis cross-talks to other pathways.

We propose that the elicitor-active compounds from the fungi activate mild defense responses to balance beneficial and none-beneficial traits in the symbiosis and to prevent over colonisation. After longer cocultivation periods, the ability of the host to control the propagation of the fungi might become weaker. [Ca^2+^] elevation occurs in the cytoplasm, a typical hallmark for Ca^2+^-induced defense responses. In beneficial arbuscular mycorrhizal symbiosis, [Ca^2+^] elevation occurs around the nuclear compartment and the signaling pathways include completely different components. Therefore, it is likely that cellotriose from *P. indica* and the unknown chemical mediators in the CW preparations from the *Fusarium* species are involved in activating immune responses. This might be comparable to the role of indole-3-acetaldoxime-derived compounds which restrict root colonization in the beneficial interaction between Arabidopsis roots and *P. indica* (Nongbri et al. 2012). PARN could be one of many components in the arms raised between the two symbionts which participates in the establishment of a balance interaction. This might be a prerequisite for the beneficial effects of the *F. incarnatum* strain K23 for their hosts under salt stress (Onejeme et al. 2024, Pallavi and Nataraja (2022).

## Open access

This article is licensed under a Creative Commons Attribution 4.0 International License, which permits use, sharing, adaptation, distribution and reproduction in any medium or format, as long as you give appropriate credit to the original author(s) and thesource, provide a link to the Creative Commons license, and indicate if changes were made. The images or other third party material in this article are included in the article’s Creative Commons license, unless indicated otherwise in a credit line to the material. If material is not included in the article’s Creative Commons license and your intendeduse is not permitted by statutory regulation or exceeds the permitteduse, you will need to obtain permission directly from the copyright holder. To view a copy of this license, visit http://creativecommons.org/licenses/by/4.0/.

## Supplementary Information

Below is the link to the electronic supplementary material.

Supplementary file1 (DOCX 13 KB)

## Data Availability

Access to all datasets is given in the mentioned publications.
