# Supplementary material for: A cell wall extract of a Fusarium incarnatum strain requires the mitochondrial POLY(A)-SPECIFIC RIBONUCLEASE AtPARN for inducing cytoplasmic calcium elevation in Arabidopsis roots
Source: Physiol Mol Biol Plants. 2025 Jul 2;31(6):851–61. doi: 10.1007/s12298-025-01600-7 (PMC12314164; doi:10.1007/s12298-025-01600-7)
Supplement: Supplementary file 1 — Supplementary file1 (DOCX 13 KB) [file 12298_2025_1600_MOESM1_ESM.docx]

**Supplementary Material**

Primers used for this study

| Gene | Forward | Reverse |
| --- | --- | --- |
| *CORK1* (At1G56145) | ACCAGAGTACGTGATGCTTG | TCCATGCCCATTCGAGAAG |
| *P. indica* *PiTEF1* | CGCAGAATACAAGGAGGCC | CGTATCGTAGCTCGCCTGC |
| *WRKY30* (AT5G24110) | CGGAGCCAAATTTCCAAGAGG | GACGGAGAGTTTGATGCTGAG |
